# Transoral robotic surgery vs open surgery in head and neck cancer. A systematic review of the literature

**DOI:** 10.4317/medoral.23632

**Published:** 2020-07-19

**Authors:** Àlvar Roselló, Rui Albuquerque, Xavier Roselló-Llabrés, Antonio Marí-Roig, Albert Estrugo-Devesa, José López-López

**Affiliations:** 1 DDS, MS. Oral Medicine, Oral Surgery and Oral Implantology. School of Dentistry. University of Barcelona, L'Hospitalet de Llobregat, Barcelona, Spain; 2 Oral Medicine Department, Guys and St Thomas NHS Foundation Trust. Faculty of Dentistry, Oral and Craniofacial Sciences, King’s College London, United Kingdom; 3 MD, DDS, PhD. Oral Medicine, Oral Surgery and Oral Implantology. School of Dentistry. University of Barcelona, L'Hospitalet de Llobregat, Barcelona, Spain; 4 MD, DDS, PhD. Oral Health and Masticatory System Group (Bellvitge Biomedical Research Institute) IDIBELL. University of Barcelona, L'Hospitalet de Llobregat, Barcelona, Spain

## Abstract

**Background:**

TORS has become one of the latest surgical alternatives in the treatment of oropharynx squamous cell carcinomas (OPSCC) and has become increasingly accepted by surgeons as a treatment option. Surgical robots were designed for various purposes, such as allowing remote telesurgery, and eliminating human factors like trembling. The study aimed to compare systematic review of the available literature in order to evaluate the safety and efficacy of Transoral Robotic Surgery (TORS) compared with open surgery.

**Material and Methods:**

We performed a systematic review of the available literature in order to evaluate the safety and effectiveness of TORS compared with open surgery. We compared TORS and open surgery based on 16 outcomes divided in to 3 groups: intra-operative complications, post-operative complications, and functional and oncologic outcomes. An electronic search of observational studies was carried out using the following databases: MEDLINE, EMBASE, Cochrane Central Register of Controlled Trials, Cochrane Oral Health Group Trials Register, and Scielo. Data analysis was carried out in accordance to Preferred Reporting Items for Systematic Reviews and Metanalysis (PRISMA) and the quality of the studies were evaluated using the Newcastle-Ottawa Scale. No language restrictions were imposed.

**Results:**

From the 4 studies identified (Newcastle-Ottawa Scale mean score 6.5), 371 patients were revised (186 patients were treated with TORS and 185 with conventional surgery). Overall, TORS, when compared with open surgery, appears to have better functional results (less hospital time, decannulation) and fewer intraoperative and post-operative complications. There is no significant difference when assessing the oncological outcomes (positive margins, survival rate) when comparing both techniques.

**Conclusions:**

TORS has an overall better functional outcome, and less intraoperative and postoperative complications with no difference in positive margins and survival rate when compared with conventional therapy.

** Key words:**Transoral Robotic Surgery, TORS, open surgery, conventional surgery, head and neck cancer, oral cancer.

## Introduction

Over the last few years, the incidence of oropharynx squamous cell carcinomas (OPSCC) associated with the Human Papilloma Virus (HPV), is significantly increasing (1-3). It is expected that if this rate of OPSCC growth continues, then by 2030 there will be at least twice as many annual cases in the U.S.A, compared to present (4). Open surgery still remains the most preferable treatment option in terms of mobility and cosmetic impact (4). Adjuvant radiotherapy (RT) with lymphnode surgery, has been used for more than three decades, but it is associated with multiple complications and reduction in patients’ quality of life (QoL) (5,6).

Chemoradiotherapy (CRT) is no longer the first-line treatment of choice, due to the demographic changes that have occurred in patients with head and neck squamous cell carcinomas (HNSCC). The type of patient currently diagnosed ranges widely, from the elderly patient with a long history of tobacco consumption and alcohol abuse, to the younger and healthier patient with HPV-associated OPSCC, having no history of substance abuse and thus a better prognosis (1,7). Although patients treated with CRT avoid surgery-associated risks (namely anaesthetic complications, bleeding, postoperative pain, fistula formation, malocclusion, facial deformities and iatrogenic lesions in neurovascular structures), they are not entirely free from complications. For example, late complications such as xerostomia, osteoradionecrosis, mucositis, nephrotoxicity, neurotoxicity, ototoxicity, sepsis, pharyngeal and oesophageal stenosis, fibrosis in muscles involved in speech and swallowing, and the development of a second malignancy have all been reported (2,4,8,9). Therefore, concerns about the toxicity of the treatment and late complications have become even more relevant, since these patients, given their age, potentially have a higher risk of developing late adverse effects (2).

Renewed interest in the search for a surgical approach for the treatment of OPSCC led to the introduction of Transoral Laser Microsurgery (TLM). In 2003, Steiner *et al*. (10) were the pioneers in publishing a series regarding this subject. TLM is a technically-challenging procedure, requiring specific training, a great deal of experience, and the results are still not entirely convincing. Additionally, the CO2 laser used is not an ideal tool for haemostasis as, during the procedure, multiple alternations are often required between the laser and a cautery instrument. Also, the fact that the microscope and laryngoscopes provide a somewhat limited view of the oropharynx with low light; may result in some surgeons feeling like they are operating in a dark hole (2,11).

Transoral Robotic Surgery (TORS) has become one of the latest surgical alternatives in the treatment of OPSCC and has become increasingly accepted by surgeons as a treatment option (11). Surgical robots were designed for various purposes, such as allowing remote telesurgery, and eliminating human factors like trembling (11). Since the introduction of robotic surgery in the field of surgery in 1985, it has been used in a range of surgical disciplines, such as orthopaedics, urology, radiosurgery, cardiac surgery and neurosurgery (12). Currently, there have been several institutional studies describing the TORS experience, but these multi-centre data results are limited (1-3). Further review of the literature, carried out by the authors, reveals a lack of evidence when assessing the qualities of TORS and Open Surgery studies. This is especially apparent when assessing the rigorous outcomes. Consequently, we aim to compare and understand the impact of TORS against conventional open surgery. We will achieve this by carrying out a systematic review of the relevant literature comparing the two techniques.

## Material and Methods

- Sources of Information and Development of Objective Questions

An electronic literature search was carried out by three reviewers (AR, RA & JLL) using various databases, including: MEDLINE, EMBASE, Cochrane Central Register of Controlled Trials, Cochrane Oral Health Group Trials Register and Scielo.

This study followed the PRISMA (Preferred Reporting Items for Systematic Review and Meta-Analyses) guidelines for a proper development of the systematic review and data analysed according to PICO (Patient, Intervention, Comparison and Outcome) (13).

- Screening process

The combination of control terms (MeSH and EMTREE) and key words were used wherever possible. The search terms used are listed below, where "[mh]" represented the MeSH terms and "[tiab]" represented a title and/or abstract: (“transoral robotic surgery” [mh] OR “TORS”[ti]) AND (open surgery [tiab]) OR (salvage surgery [tiab]) OR (conventional surgery [tiab]) AND (head and neck cancer [tiab]) OR (oral cancer [tiab]). In addition, a manual search was carried out of the journals related to head and neck oncology to ensure a thorough selection process. We applied no language restrictions. The references in the excluded articles were also checked by seeking studies that met the inclusion criteria. Articles published in the last 6 years, up to December 2019 were reviewed.

- Eligibility Criteria

**Figure 1:**
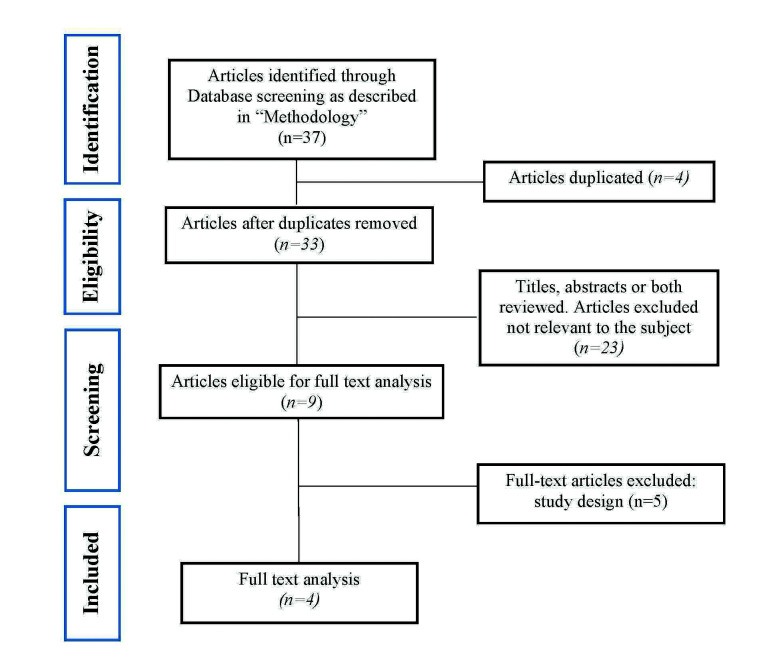
PRISMA flow chart.

The full papers and abstracts identified through the search engines were independently reviewed by the authors (AR, RA & JLL) for inclusion in this systematic review. If there was a disagreement between the reviewers, the authors reviewed the full text before reaching an agreement through discussion. Data extraction was then completed in duplicate by the same independent reviewers. The search strategy and the flow diagram of the article selection are shown in Fig. 1.

The articles that were included in this systematic review met the following inclusion criteria: retrospective or prospective clinical observational studies, with 50 or more human subjects and the clinical results of TORS in head and neck cancer were reported. Consequently, several factors such as study design, number of patients included in the latest follow-up assessment, test/control groups, tumour size and TNM staging were studied. Furthermore, in order to address the aim of this study more comprehensibly, other technique-related parameters were also extracted, including operating time, blood loss, postoperative complications (e.g. Infection, bleeding, and respiratory tract oedema), functional outcomes (e.g. duration of in-patient stay, and swallowing function) and oncological outcomes (average survival and disease-free average). By contrast, case reports or case series, studies comparing TORS with Chemoradiotherapy (CRT), reviews, author debates, letters to the editor, pre-clinical studies, clinical studies involving animals or with deficient information were excluded.

- Quality of studies

The Newcastle-Ottawa Scale (NOS) was used to assess the quality of non-randomised studies (Case Controls & Cohorts Studies) by 5 reviewers (AR, XRL, AMR, EJS, JLL). Using a scoring system with stars, each study was judged on three broad aspects: the selection of study groups, the homogeneity of the compared groups and the methodology of the results obtained (Table 1). If there was a disagreement between the reviewers, the authors would reach a consensus and decide the overall score.

## Results

- Selection of articles

An initial assessment obtained 33 articles, of which 10 were selected that were potentially relevant after evaluating their abstracts (1,3,6,9,14-19). The full texts of those 10 articles were then obtained. Of these, 4 articles (3,6,9,14) met the inclusion criteria and they were analysed qualitatively (Fig. 1).

- Results of the systematic review

The total number of patients studied was 371 (305 men and 66 women). 186 patients were treated with TORS and 185 with conventional surgery. With regard to the tumour staging, there were 118 cases of T1, 194 at T2, 41 at T3 and 18 at T4. Regarding TNM staging, there were a total of 50 cases of TNM I, 60 cases of TNM II, 50 cases of TNM III and 211 cases of TNM IV (Table 2).

Two of the articles (6,14) referred to the amount of blood lost during surgery, with a finding of a difference of over 200ml between the control group (open surgery) and the study group (TORS). 3 of the 4 articles (6,9,14) also discussed the operating times and a noTable difference can be seen between the shortest times in the study group than in the control group. However, it should be mentioned that in the article by Lee *et al*. (9) there is a subgroup in the test group which had much longer intraoperative times due to the need to perform mandibulectomy (Table 3).

**Table 1:**
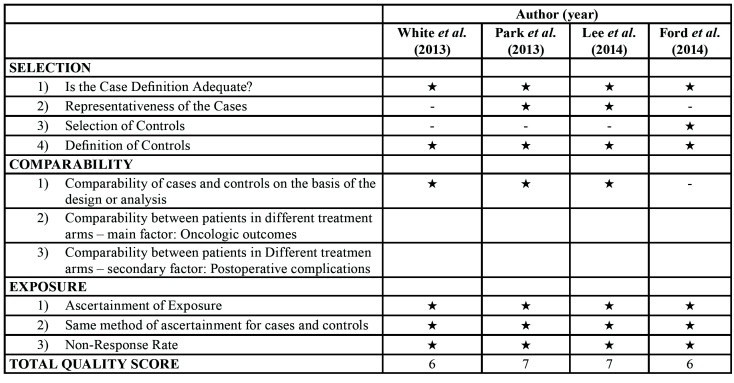
Quality assessment of selected studies based on Newcastle-Ottawa Scale.

Among the postoperative complications, it is noted that 3 out of the 4 articles do not obtain statistically significant results on the positive surgical margins, since the results of this are very similar between the two groups. Only the article by White *et al*. (6) obtained significant results, since the TORS group only had 6 cases of positive margins and the open surgery group had 19 cases. In terms of postoperative bleeding, the studies showed better results in the study group; Park *et al*. (14) obtained 3.33% in the TORS group compared with 19.23% in the open surgery group and White *et al*. (6) obtained 11% in the test group against 13% in the control group. With regards to postoperative fistulas, only two articles discussed these. The two studies had a very similar percentage of fistulas: 6% (6) and 7.69% (14). However, in neither of the two studies do the fistulas occur in the TORS group, but in the open surgery group (Table 3).

As regards the functional results, it can be seen that TORS obtained significant results compared with open surgery (Table 4). The hospitalisation time varies a lot between the various studies, but among them all, the TORS group reveals a better result; 3.8 days (6), 26.1 days (14) and 14.6 days (9) against 8.0 days (6), 43.4 days (14) and 24.6 days (9), respectively. Tracheotomy cannot be assessed properly since not all study groups followed the same criteria - some studies performed it in advance (14) and others did not (6,9). However, in decannulation we observed that the TORS group obtained much better results. Park *et al*. (14) took an average of 7.2 days to decannulate TORS patients and 15 days in open-surgery patients. Likewise, Lee *et al*. (9) took an average of 5 days in the TORS group and 13.2 in the control group. We can also observe that patients treated with TORS regained their swallowing ability much quicker; 8.4 (14) and 6.5 days (9) on average compared with 20.6 (14) and 16.5 (9) days in patients treated with open surgery.

**Table 2:**
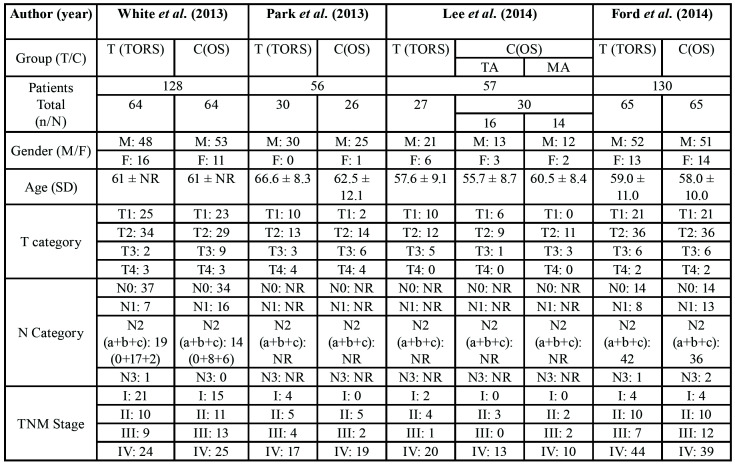
Patients treated with the TORS technique an conventional technique according to Gender, Age, T Category, N Category, TNM Stage. C, group control; F, female; M, male; MA, mandibulotomy approach; NR, not reported; OS, open surgery; T, group test; TA, transoral approach; TORS; transoral robotic surgery.

**Table 3:**
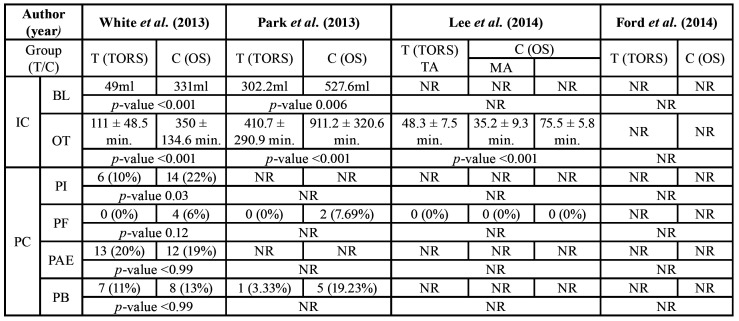
Patients treated with the TORS technique and conventional technique according to Intraoperative and Postoperative complications. BL, Blood loss; C, group control; F, female; IC, intraoperative complications; M, male; MA, mandibulotomy approach; Ml, millilitres; Min, minutes; NR, not reported; OT, operative time; OS, open surgery; PAE, postoperative airway edema; PB, postoperative bleeding; PF, postoperative fistula; PI, postoperative infection; T, group test; TA, transoral approach; TORS; transoral robotic surgery.

**Table 4:**
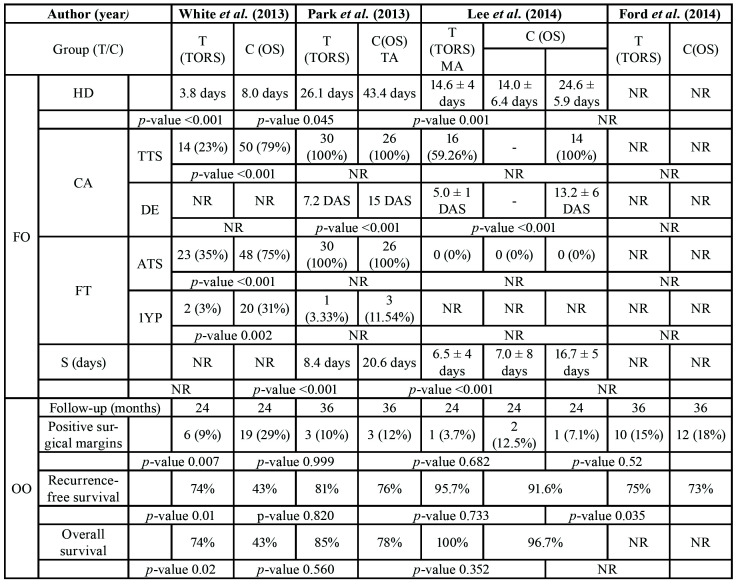
Patients treated with the TORS technique and conventional technique according to Functional and Postoperative complications. 1YP, 1 year postoperative; ATS, At the time of surgery; C, group control; CA, cannulate; DAS, days after surgery; DE, Decannulated; F, female; FT, Feeding tube; FO; functional outcomes; FU(M), Follow-up (months); HD, hospital day; M, male; MA, mandibulotomy approach; NR, not reported; OO, Oncologic Outcomes; OS, open surgery; S, Swallowing; T, group test; TA, transoral approach; TORS; transoral robotic surgery; TTS, Tracheostomy use at the time of surgery.

With regards to the oncological results, 3 (3,9,14) out of the 4 articles (3,6,9,14) do not show any significant results in terms of disease-free and survival time, and the differences between the test and control groups were very similar. Furthermore, the study by White *et al*.(6) shows significant results in both disease-free time (74% test group, 43% control group) and survival (74% test group, 43% control group) (Table 4).

- Quality of Studies

The Newcastle-Ottawa Scale (NOS) was used to analyse the quality of observational studies and scores of between 6 and 7 were obtained (Table 1).

## Discussion

The nature of treating head and neck cancer raises several ethical reasons when conducting randomised clinical trials. Consequently, evidence for TORS and open surgery to treat head and neck cancer is limited. Assessing the quality of observational studies allows us to address some of previous limitations. Although, we acknowledge the limitations commonly seen at cohort and case control studies selected for this study.

There are several methods to address the quality of evidence for observational studies such as ROBINS, Newcastle-Ottawa Scale (NOS), “Downs and Black” and “Scottish Intercollegiate Guidelines Network’s methodology checklist”. Although ROBINS has a has been highlighted, for its good validity (20), when assessing surgical records NOS is easy to apply (21), and has been widely used due to recommendations from the Cochrane Collaboration (22). Newcastle-Ottawa Scale (NOS) has been used in other systematic reviews to assess TORS2 but has not yet been compared with open surgery. In order to provide similar methodology, as well as reveal the advantages previously mentioned, we agreed to use NOS. However, we do acknowledge the limitations of NOS, when it is compared with ROBINS.

Treating HNSCC patients with surgery, instead of CRT, means they avoid the typical complications of RT and CT; xerostomia, osteoradionecrosis, mucositis, sepsis and trismus (2,4,9,23). A recent study (24) reported an overall survival rate of 84.9% and a low rate of xerostomia in 442 patients who received intense modulated radiotherapy. However, Forastiere *et al*. (25) reported that toxicity increased by combining chemotherapy and radiotherapy, causing chronic lesions and severe fibrosis in the pharyngeal mucosa and masticatory mucosa. Whilst treating patients with surgery prevents them from experiencing complications of CRT, they are not free from complications. Some previous studies indicate that almost 50% of patients who undergo open surgery for cancers of the head and neck develop postoperative complications or problems with the healing of wounds (26).

Surgical complications of TORS include: formation of fistulas, postoperative haemorrhages, as well as surgical infections, pneumonia, nerve damage and lesions of the teeth (27). Two studies conducted at the Mayo Clinic (Rochester, MN, USA.) claim that their series of case studies resulted in 6% formation of fistulas (28,29). These patients underwent lymphadenectomy at the same time as TORS surgery. Fistulas were resolved with antibiotic treatment. Chia *et al*. (30) in a retrospective survey only had 0.2% of cases with fistulas in patients who received TORS, which is very close to the results of our review. As mentioned previously, only two studies (6,14) provided data regarding fistulas, but all instances occurred in cases of open surgery. None of the cases treated with TORS developed fistulas. Blood loss, prior chemotherapy and type of surgery are risk factors for postoperative infections and fistulas (31).

Postoperative haemorrhage is a potentially life-threatening complication in patients treated with TORS. In the same study by Chia *et al*. (30) it is reported that, although the mortality rate after TORS was low (0.3%), haemorrhage was the causative factor in of all cases. Doazan *et al*. (32) reported 2 deaths because of postoperative hemorrhage (1.6%). This rate is higher than what was reported for the series of Mandal *et al*. (33) with 0.9% of severe hemorrhage that led to death. A total of 3.1% of postoperative haemorrhages were reported which closely approximates the analysed study by Park *et al*. (14), which has a haemorrhage rate of 3.3%, which are values that approximate a very recent published review, with a rate of 2.4% (34). However, some articles record values close to 6% (35) or even above 10%, such as the analysed study by White *et al*., with 11% or the multi-centre study by Aubry *et al*. (36) who reported18.5% of postoperative haemorrhages. As expected, Asher *et al*. (35) found that larger tumours have a greater bleeding risk than smaller tumours. Anticoagulant therapy and/or anti-platelet therapy was the only other risk factor identified in postoperative haemorrhages (35,36). Based on these results, additional data and larger samples are needed to obtain more accurate conclusions with regards to postoperative haemorrhages. However, given the results of this review, intraoperative bleeding is much less when using TORS than open surgery; but in postoperative bleeding there is not much difference between the two types of surgery and there is even a risk of death due to postoperative bleeding (30). While the surgical robot is an excellent tool to improve visualisation and instrumentation in the upper digestive tract, unfortunately, TORS can be less ideal for controlling haemorrhages after transoral robotic surgery. This reflection led Asher *et al*. (35) to perform preventive ligatures of the lingual artery. Improvements are being attempted in this respect, such as: the use of intraoperative ultrasound images in real time to increase the 3D visualisation of the robotic system. Ultrasound imaging may allow the identification of the tumour boundaries and neurovascular structures, thus improving tumour resection and reducing morbidity (36).

TORS provides the ability to carry out, under a 3D-HD view enlarged on multiple planes, en-bloc tumour resections, allowing the margins to be assessed more accurately during resections. The greater degree of instrumentation freedom, means that the safety margins of resections have results very similar to those of conventional open surgery, but with a much lower cost in terms of surgical morbidity (37). Our systematic review confirms this, since out of the 4 articles analysed, 3 showed no significant results regarding safety margins. Only the study by White *et al*. (6) reported significant results, resulting in 9% positive margins in the TORS group and 29% in the control group. The results on the safety margins are likely to have a direct proportional relationship to the overall survival rates of the disease and disease-free patients, since only the same study by White *et al*. (6) presents statistically significant results in these two sections. However, we must also note that the great difference between the results of White *et al*. (6) and those of the other 3 studies (3,9,14) on these two indices. The other 3 studies (3,9,14) obtained between 76-96.7% in the control groups in these indices and the study by White *et al*. (6) only obtained 43%, in both the patient survival rate and in disease-free time in the open surgery group. One explanation could be that the TORS group only had 5 patients with T3 or T4, while the open surgery group had a total of 12 patients. The larger the tumour, the higher the rate of positive margins.

A multi-centre study (38), with 410 patients, who were treated with TORS, obtained loco-regional control rates of 91.2% at 24 months and of 88.8% at 36 months. The results of this study are very similar to the results obtained by the studies analysed in this review, reinforcing the idea the TORS has high rates of survival and disease-free patients.

From a functional point of view, many clinical studies have shown an improved swallowing function in patients treated with TORS, compared with other surgical methods and even compared with primary chemotherapy. They also show a much shorter hospital stay and faster recovery (27). In terms of function we can state that TORS demonstrates more beneficial results than open surgery. All of the studies analysed obtained significant results in the reduction of time spent in hospital and in the better swallowing ability of patients treated with TORS.

Another aspect to be noted is the cost-effectiveness analysis of TORS versus open surgery. Dombrée *et al*. (39) compared TORS, TLM and open surgery for partial and total laryngectomies. Interestingly enough, TLM and open surgery obtained better results against TORS; maybe a reason for these results is the high initial investment needed for TORS. Another recent article (40) concluded that TORS cost are 24-35% less than IMRT, but that one of the reasons why TORS costs could increase is the adjuvant therapy as de Almeida *et al*. (38) explains. Spellman *et al*. (40) concluded that TORS is highly cost-effectiveness is early stage oropharyngeal cancer.

One of the limitations of the analysed studies is that they are rather heterogeneous with one another: Ford *et al*. (3) and White *et al*. (6) studied salvage surgery for OPSCC while Park *et al*. (14) studied salvage surgery, in hypopharyngeal SCC, and Lee *et al*. (9) studied only T1-T3 in tonsillar cancer. Results of this systematic review should therefore be analysed with caution. The multiple co-founders, which are difficult to control outside of a clinical trial, should be taken into account and consequently the level of evidence should be interpreted as limited.

## Conclusions

In conclusion, TORS appears to be a promising technique with fewer complications compared with open surgery. Its effectiveness depends on the location of the tumour, for which more specific studies of each type of cancer are needed. The skill of the surgical team also has an effect, since the training curve is relevant. TORS obtains much better functional results when compared with Open Surgery therapeutic techniques for treating patients with HNSCC. Although the oncological outcomes of TORS are good, they do not appear better than those of open surgery.

We suggest that more high quality observational studies, to understand the effectiveness of TORS when compared with Open Surgery, are needed. The studies will need to use good statistical power and standardised outcome measures to obtain actuate results.

## References

[1] Nichols AC, Yoo J, Hammond JA, Fung K, Winquist E, Read N (2013). Early-stage squamous cell carcinoma of the oropharynx: radiotherapy vs. trans-oral robotic surgery (ORATOR)--study protocol for a randomized phase II trial. BMC Cancer.

[2] Kelly K, Johnson-Obaseki S, Lumingu J, Corsten M (2014). Oncologic, functional and surgical outcomes of primary Transoral Robotic Surgery for early squamous cell cancer of the oropharynx: A systematic review. Oral Oncol.

[3] Ford SE, Brandwein-Gensler M, Carroll WR, Rosenthal EL, Magnuson JS (2014). Transoral robotic versus open surgical approaches to oropharyngeal squamous cell carcinoma by human papillomavirus status. Otolaryngol Head Neck Surg.

[4] Mercante G, Masiello A, Sperduti I, Cristalli G, Pellini R, Spriano G (2015). Quality of life and functional evaluation in patients with tongue base tumors treated exclusively with transoral robotic surgery: A 1-year follow-up study. J Craniomaxillofac Surg.

[5] Dziegielewski PT, Teknos TN, Durmus K, Old M, Agrawal A, Kakarala K (2013). Transoral robotic surgery for oropharyngeal cancer: long-term quality of life and functional outcomes. JAMA Otolaryngol Head Neck Surg.

[6] White H, Ford S, Bush B, Holsinger F, Moore E, Ghanem T (2013). Salvage surgery for recurrent cancers of the oropharynx: comparing TORS with standard open surgical approaches. JAMA Otolaryngol Head Neck Surg.

[7] Chang C, Chang S, Chuang S, Berthiller J, Ferro G, Matsuo K (2019). Age at start of using tobacco on the risk of head and neck cancer: Pooled analysis in the International Head and Neck Cancer Epidemiology Consortium (INHANCE). Cancer Epidemiol.

[8] Byrd JK, Ferris RL (2016). Is There a Role for Robotic Surgery in the Treatment of Head and Neck Cancer?. Curr Treat Options Oncol.

[9] Lee Sei Y, Park Min Y, Byeon Kwon H, Choi Chang E, Kim S H (2014). Comparison of oncologic and functional outcomes after transoral robotic lateral oropharyngectomy versus conventional surgery for T1 to T3 tonsillar cancer. Head Neck.

[10] Steiner W, Fierek O, Ambrosch P, Hommerich CP, Kron M (2003). Transoral laser microsurgery for squamous cell carcinoma of the base of the tongue. Arch Otolaryngol Head Neck Surg.

[11] Moore EJ, Hinni ML (2013). Critical review: Transoral laser microsurgery and robotic-assisted surgery for oropharynx cancer including human papillomavirus-related cancer. Int J Radiat Oncol Biol Phys.

[12] De Ceulaer J, De Clercq C, Swennen GRJ (2012). Robotic surgery in oral and maxillofacial, craniofacial and head and neck surgery: A systematic review of the literature. Int J Oral Maxillofac Surg.

[13] Moher D, Liberati A, Tetzlaff J, Altman DG (2009). Preferred reporting items for systematic reviews and meta-analyses: the PRISMA statement. Ann Intern Med.

[14] Park Min Y, Byeon Kwon H, Chung Pil H, Choi Chang E, Kim S H (2013). Comparison study of transoral robotic surgery and radical open surgery for hypopharyngeal cancer. Acta Otolaryngol.

[15] More YI, Tsue TT, Girod D, Harbison J, Sykes K, Williams C (2013). Functional swallowing outcomes following transoral robotic surgery vs primary chemoradiotherapy in patients with advanced-stage oropharynx and supraglottis cancers. JAMA Otolaryngol Head Neck Surg.

[16] de Almeida JR, Moskowitz AJ, Miles BA, Goldstein D, Teng M, Sikora A (2014). Cost-effectiveness of transoral robotic surgery versus (chemo)radiotherapy for early T classification oropharyngeal carcinoma: A cost-utility analysis. Head Neck.

[17] Chen AM, Daly ME, Luu Q, Donald PJ, Farwell DG (2015). Comparison of functional outcomes and quality of life between transoral surgery and definitive chemoradiotherapy for oropharyngeal cancer. Head Neck.

[18] de Almeida JR, Villanueva NL, Moskowitz AJ, Miles B, Teng M, Sikora A (2014). Preferences and utilities for health states after treatment for oropharyngeal cancer: transoral robotic surgery versus definitive (chemo) radiotherapy. Head Neck.

[19] Slama K, Slouka D, Slipka J, Fischer S (2016). Short-term postoperative distress associated with open vs. Transoral robotic surgery (TORS) in patients with T1-T2 carcinomas of the tongue base and supraglottis. Biomed Pap.

[20] Sterne JAC, Hernán MA, Reeves BC, Savovíc J, Berkman N, Viswanathan M (2016). ROBINS-I : a tool for assessing risk of bias in non-randomised studies of interventions. BMJ.

[21] Deeks JJ, Dinnes J, D'Amico R, Sowden A, Sakarovitch C, Song F (2003). Evaluating non-randomised intervention studies. Health Technol Assess.

[22] Downs SH, Black N (1998). The feasibility of creating a checklist for the assessment of the methodological quality both of randomised and non-randomised studies of health care interventions. Journal of Epidemiology and Community Health.

[23] Watters AL, Cope S, Keller MN, Padilla M, Enciso R (2019). Prevalence of trismus in patients with head and neck cancer: A systematic review with meta-analysis. Head Neck.

[24] Setton J, Caria N, Romanyshyn J, Koutcher L, Wolden S, Zelefsky M (2012). Intensity-Modulated Radiotherapy in the Treatment of Oropharyngeal Cancer: An Update of the Memorial Sloan-Kettering Cancer Center Experience. Int J Radiat Oncol • Biol • Phys.

[25] Forastiere AA, Trotti A (1999). Radiotherapy and concurrent chemotherapy: a strategy that improves locoregional control and survival in oropharyngeal cancer. J Natl Cancer Inst.

[26] Zafereo ME, Hanasono MM, Rosenthal DI, Sturgis E, Lewin J, Roberts D (2009). The role of salvage surgery in patients with recurrent squamous cell carcinoma of the oropharynx. Cancer.

[27] Gorphe P (2018). A Contemporary Review of Evidence for Transoral Robotic Surgery in Laryngeal Cancer. Front Oncol.

[28] Moore EJ, Olsen KD, Kasperbauer JL (2009). Transoral robotic surgery for oropharyngeal squamous cell carcinoma: a prospective study of feasibility and functional outcomes. Laryngoscope.

[29] Moore EJ, Olsen SM, Laborde RR, García J, Walsh F, Price D (2012). Long-term functional and oncologic results of transoral robotic surgery for oropharyngeal squamous cell carcinoma. Mayo Clin Proc.

[30] Chia SH, Gross ND, Richmon JD (2013). Surgeon experience and complications with Transoral Robotic Surgery (TORS). Otolaryngol Neck Surg.

[31] Ogihara H, Takeuchi K, Majima Y (2009). Risk factors of postoperative infection in head and neck surgery. Auris Nasus Larynx.

[32] Doazan M, Hans S, Morinière S, Lallemant B, Vergez S, Aubry K (2018). Oncologic outcomes with transoral robotic surgery for supraglottic squamous cell carcinoma: Results of the French Robotic Surgery Group of GETTEC. Head Neck.

[33] Mandal R, Duvvuri U, Ferris RL, Kaffenberger TM, Choby GW, Kim S (2016). Analysis of post-transoral robotic-assisted surgery hemorrhage: Frequency, outcomes, and prevention. Head Neck.

[34] Helman SN, Schwedhelm T, Kadakia S, Wang Y, Schiff BA, Smith R V (2015). Transoral Robotic Surgery in Oropharyngeal Carcinoma. Arch Pathol Lab Med.

[35] Asher SA, White HN, Kejner AE, Rosenthal EL, Carroll WR, Magnuson JS (2013). Hemorrhage after transoral robotic-assisted surgery. Otolaryngol Head Neck Surg.

[36] Aubry K, Vergez S, de Mones E, Morinière S, Choussy O, Malard O (2016). Morbidity and mortality revue of the French group of transoral robotic surgery: a multicentric study. J Robot Surg.

[37] Genden EM, O'Malley BW, Weinstein GS, Stucken CL, Selber JC, Rinaldo A (2012). Transoral robotic surgery: Role in the management of upper aerodigestive tract tumors. Head Neck.

[38] de Almeida JR, Li R, Magnuson JS, Smith S, Moore E, Lawson G (2015). Oncologic Outcomes After Transoral Robotic Surgery: A Multi-institutional Study. JAMA Otolaryngol Head Neck Surg.

[39] Dombrée M, Crott R, Lawson G, Janne P, Castiaux A, Krug B (2014). Cost comparison of open approach, transoral laser microsurgery and transoral robotic surgery for partial and total laryngectomies. Eur Arch Otorhinolaryngol.

[40] Spellman J, Coulter M, Kawatkar A, Calzada G (2020). Comparative cost of transoral robotic surgery and radiotherapy (IMRT) in early stage tonsil cancer. Am J Otolaryngol.

